# A Simplified “One-Pathway” Protocol for the Transformation of General Wards to Infectious Disease Wards Upon the Pandemic Outbreak

**DOI:** 10.3389/fpubh.2022.894669

**Published:** 2022-06-24

**Authors:** Jikai Yin, Dongdong Li, Meijuan Peng, Wangang Guo

**Affiliations:** ^1^The 2nd Hubei Medical Aid Team, Tangdu Hospital, Air Force Medical University, Xi'an, China; ^2^Department of General Surgery, Tangdu Hospital, Air Force Medical University, Xi'an, China; ^3^Department of Cardiology, Tangdu Hospital, Air Force Medical University, Xi'an, China

**Keywords:** COVID-19, infectious disease wards, general hospital, working protocol, reconstruction

## Introduction

In 2020, the medical systems of various countries worldwide have been unexpectedly impacted and overwhelmed by the coronavirus disease 2019 (COVID-19) pandemic (1, 2). Hospitals and wards for infectious diseases fell short of demand (3). Temporary compartment hospitals were designated for patients with mild symptoms or asymptomatic infection, while numerous patients with severe symptomatic COVID-19, even those with complications, could not be quarantined and treated in infectious disease wards (4). Therefore, it was an urgent need to transform the existing general wards into temporary infectious disease wards for patients with COVID-19. However, general wards had limitations to meet the structural criteria requiring “three areas, two passages, and two hallways” for receiving patients with infectious disease (5). The “three areas” refer to “contaminated,” “potentially contaminated,” and “clean” areas. The “two passages” refer to staff entering and exiting passages. The “two hallways” refer to the patient and staff-only hallways. This classic structure has been proved to be safe and effective in Ebola treatment centers (6). General hospitals involved in epidemic control were facing an urgent challenge of finding a simple and fast transformation protocol, which would be compliant with the infection control policy of China.

**Figure 1:**
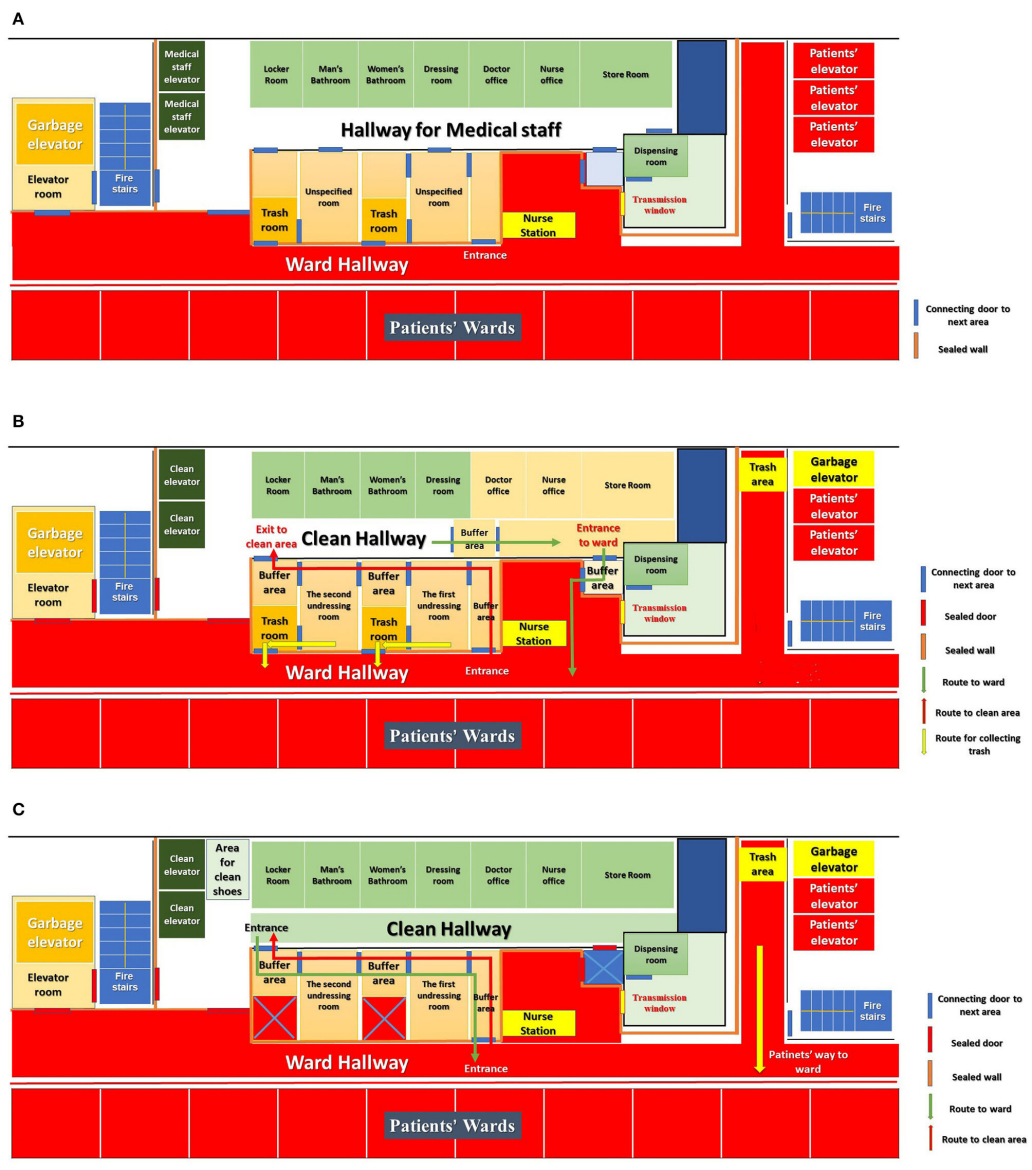
Transformation of general wards into infectious disease wards. **(A)** The original plane graph of the general wards in the general hospital. **(B)** The standard reconstruction (STR) and working protocol of “Two-Pathway” transforming the general ward into infectious disease ward. **(C)** The simplified reconstruction (SIR) and working protocol of “One-Pathway” transforming the general ward into infectious disease ward.

All authors of the present article are from Tangdu Hospital, which was designated to receive patients with confirmed COVID-19 in Shaanxi Province during the COVID-19 outbreak (7). This hospital also dispatched medical teams to Wuhan to work in temporary COVID-19 hospitals and treat the patients with confirmed COVID-19. Most of the authors of this article worked in the Maternity and Child Healthcare Hospital of Hubei province since February 17, 2020. Figure 1A shows the original plane graph of the general wards in this hospital, whose design is quite universal for Chinese public hospitals. Three groups of elevators are set in different areas for patients, medical staff, and waste delivery. There were two fire stairs located beside patient and staff elevator rooms. The office area and the ward were independent of each other. The medical staff passed through a simple and independent passage and doors to enter or exit the ward. Patients and their family members could not freely enter the office area.

## The Standard Reconstruction Protocol and Its Disadvantages

The standard reconstruction (STR) protocol (Figure 1B) was used trying to set up “Three Areas and Two Passages”, thus, transforming the general wards into the infectious disease wards. The “Two Hallways” could not be realized in public hospitals. The office area was separated into two areas, i.e., the clean area (green area, GA) and the potentially contaminated area (yellow area, YA). The whole ward and ward hallway were regarded as the contaminated area or red area (RA). With such a setup, there was no second hallway for medical staff due to the limitation of the original design of general wards. The YA was supposed to be linked with the RA *via* a small buffering room with one-way doors, while the GA was supposed to be connected to the YA *via* another small buffering room with one-way doors as well. According to the STR protocol, medical staff in the GA should wear the Level-1 personal protective equipment (PPE), i.e., an N95 mask and white coat to perform management, command, and coordination work. Those in the YA should wear the Level-2 PPE, which included an N95 mask, goggles, and a medical protection jumpsuit. Staff would stay in YA to accomplish medical records and nursing management activities, such as order management and drug dispensing. Staff could enter the RA through the ward entrance buffering room, wearing the Level-3 PPE, including a face shield, an outer waterproof isolation suit, and protective boots adding to the Level-2 PPE. Due to the respiratory transmission feature of COVID-19 and infection control needs, the buffer rooms in YA were vitally important in any reconstruction protocol for air precipitation, and meanwhile, the medical staff equipped with a heavy layer of PPE also need multiple rooms and sufficient time to follow critical steps of removing their PPE. Staff in RA would need to pass at least two undressing rooms and corresponding buffering rooms for disinfection, and then, undress before entering the GA. If staff from the YA wanted to enter the GA, they would also need to enter RA before undressing. The most prominent advantage of STR was that such design offered enough space for the disinfection and sedimentation of pathogens when medical staff entered or exited the infectious disease ward. Yet, after an on-site investigation, we found that the standard “three areas and two passages” protocol were difficult to achieve in general hospitals because it was impossible to set up so many standard buffering areas in the general ward. Under the circumstances of PPE shortage (8), the additional burden would be placed on PPE supply following such protocol.

As shown in Figure 1B, such reconstruction and working protocol have at least four disadvantages: (1) The initial entering pathway only has one buffering room separating the RA and YA, which is not enough to isolate airflow from RA. YA could be contaminated by each entry into RA, which might be extremely dangerous to all medical staff working in YA; (2) Medical waste from the rooms in the undressing passage was delivered to the ward through the waste passage in each room. These waste passages could further increase the contamination possibility of undressing rooms by opening and closing doors, and through any doors accidentally left unclosed; and (3) Medical staff must wear Lever-2 PPE in YA dealing with paperwork and nursing management activities, which tremendously increased the consumption of PPE.

## “One Pathway” Protocol and Its Advantages

We hereby carried out a simplified reconstruction (SIR) protocol setting up “One Pathway” to meet the quarantine requirements for respiratory infectious diseases. There were only two areas (RA and GA) in our solution. The entrance buffering room and doors would all be closed and sealed, thus, turning YA in STR protocol into GA so as to no direct communication with RA. The unnecessary connecting doors in the ward were to be blocked, as shown in Figure 1C. The doors and buffering room between GA and YA in STR protocol could be eliminated. Consequently, the whole office area would become a fully independent GA. In this design, the medical staff would only need to wear Level 1 PPE while dealing with paperwork and nursing management activities. The “One Pathway” implies that staff enters and exits the ward through one route, i.e., the undressing and corresponding buffering rooms. Medical staff would need to be put on Level-3 PPE in GA before entering the undressing passage and ward. The passage would always be disinfected by 24-h ultraviolet lights and static electricity absorptive air sterilizer to minimize the risk of cross-infection between different rooms. Furthermore, we modified our working protocol according to the “One Pathway” structure. While entering the ward through the pathway, medical staff would be required to finish the following assignments: (1) carrying medical items, medicines, and other necessities into the RA; (2) checking the consumption of sterilizing supplies, including hand sanitizer, moist towelettes, and paper towels, in the undressing room and reminding the next shift to replenish the supply in time; (3) collecting waste in each undressing room and carrying it to RA for centralized delivery and processing. Including dressing the level-3 personal protective equipment and finishing the assignments, such as carrying the necessities, checking the sterilizing supplies, and collecting the wastes, ~15 min were needed for one entry.

Once proposed, this reconstruction and working protocol have been widely accepted by all the experts from the Center for Disease Control of China and infection control staff at our temporary hospital for five main reasons: (1) The reconstruction was simple, executable, and achievable in 2 days. It saved a lot of time because there was no need to create an entrance buffering room, trash room, additional storage room, and dressing room. During the epidemic, saving the reconstruction time means that the hospital could be immediately put into operation, thus, saving more lives, and improving the epidemic control; (2) The office area of the medical staff was greatly expanded. Only one storage room and one dressing room were needed, and buffering rooms were also greatly reduced because the YA was removed; (3) At least 50% of PPE was preserved with the SIR protocol compared to the STR protocol, as staff in GA did not need to wear Level-2 PPE doing paperwork and nursing management work; (4) The labor force was greatly reduced as to the SIR working protocol because there was no need to arrange any shifts to collect waste from undressing rooms. Staff did not need to wear Level-2 PPE while doing paperwork and nursing management, so that their shift lasted up to 8 h other than 4 h, thus, greatly reducing working personnel requirement to about 4,200 suits weekly; and (5) This reconstruction and working protocol effectively prevented air in the undressing room and GA from being contaminated by that in RA, because the only pathway connecting RA and GA was the undressing passage. The two buffering rooms and two undressing rooms were adequate for disinfection and sedimentation of pathogens in this passage. On Apr. 13, 2021, this protocol was recommended by the National Health Commission of the People's Republic of China in the “*Guideline of COVID-19 Control and Prevention Technique for Medical Institutions (2nd version)*”. Now, it has been applied by most of hospitals charged for COVID-19 quarantine and treatment of patients with COVID-19 in China.

During the outbreak of COVID-19, the admission of confirmed patients not only posed higher requirements for epidemic control facilities but also put heavier mental stress on everyone. This was another critical advantage of this reconstruction and working protocol due to a lack of medical staff during that period. The ratio of medical staff to patients was nearly 1:1 for more than 50 days when our temporary hospital received confirmed patients in Wuhan. It was a great challenge to set up a workforce-saving strategy for all medical staff, especially for nurses under such circumstances (9). The infection risk of medical staff was minimized when SIR protocol was applied for the transformation of a general hospital into an infectious disease hospital. The long undressing passage also gave all medical staff enough confidence to protect themselves (10). This might be an optimal solution for public hospitals to respond to the pandemic by receiving infectious patients quickly. A total of 1,765 confirmed patients were treated in our temporary hospital in Wuhan in 50 days. No hospital-acquired infection of medical staff occurred. After the evacuation, all the medical staff was quarantined for 14 days, and no infection or asymptomatic infection was identified by the two nucleic acid tests and the COVID-19 antibody test, supporting the rationality of this reconstruction and working protocol under such circumstances.

The potential risks of the SIR still exist in two aspects. All the medicines and devices need to be carried by staff through the “One Pathway”, which might not be timely for treatment requirements. People coming into and out of RA might meet in the undressing rooms, leading to possible cross-contamination. We could avoid such meetings by intercoms.

## Conclusion

The “One Pathway” protocol, which has been applied in the COVID-2019 outbreak in Wuhan, is simple, safe, and easy to achieve to transform a general ward into a temporary infectious disease ward. This might provide an exemplary transformation protocol for general hospitals during any alike pandemic outbreak to cope with infectious disease hospital shortages.

## Author Contributions

JY and DL wrote the manuscript. WG, JY, and MP designed and mapped the concepts. All authors contributed to the article and approved the submitted version.

## Conflict of Interest

The authors declare that the research was conducted in the absence of any commercial or financial relationships that could be construed as a potential conflict of interest.

## Publisher's Note

All claims expressed in this article are solely those of the authors and do not necessarily represent those of their affiliated organizations, or those of the publisher, the editors and the reviewers. Any product that may be evaluated in this article, or claim that may be made by its manufacturer, is not guaranteed or endorsed by the publisher.

## Abbreviations

COVID-19: coronavirus disease 2019
STR: standard reconstruction
SIR: simplified reconstruction
PPE: Personal protective equipment
GA: green area
YA: yellow area
RA: red area.
